# Sedentary behaviors and physical activity of the working population measured by accelerometry: a systematic review and meta-analysis

**DOI:** 10.1186/s12889-024-19449-y

**Published:** 2024-08-06

**Authors:** Sungwon Park, Sueyeon Lee, Seoyoon Woo, Katelyn Webster-Dekker, Weiyun Chen, Philip Veliz, Janet L. Larson

**Affiliations:** 1 School of Nursing, University of Michigan, 400 North Ingalls Street, Ann Arbor, MI 48109 USA; 2 Michigan Society of Fellows, University of Michigan, Ann Arbor, MI USA; 3 Marcella Niehoff School of Nursing, Loyola University Chicago, Chicago, IL USA; 4 School of Nursing, University of North Carolina Wilmington, Wilmington, NC USA; 5 School of Nursing, Indiana University, Indianapolis, IN USA; 6 School of Kinesiology, University of Michigan, Ann Arbor, MI USA

**Keywords:** Exercise, Sedentary Behavior, Occupational Health, Occupational Groups

## Abstract

**Background:**

Too much sedentary behavior (SB) and too little physical activity (PA) place adult workers at risk for chronic illness. It remains unclear which occupations and subgroups within occupations have the highest and lowest SB and PA, and little is known about the effects of organizational factors on these behaviors and metrics. Thus, our main aims were to review and summarize evidence describing daily SB and PA collected using accelerometry across various occupations and to identify organizational factors influencing SB and PA.

**Methods:**

A literature search of six databases was performed for relevant studies published through March 2023. Eligible studies were in English, targeted working populations, had a sample size > 75, and objectively measured both SB and PA for seven consecutive days using accelerometers. Following PRISMA guidelines, 5,197 studies were identified, and 19 articles met our inclusion criteria. Five of these studies were included in a meta-analysis comparing time spent in SB, light PA (LPA), and moderate to vigorous PA (MVPA) across occupations. Methodological quality was assessed using a Joanna Briggs Institute tool.

**Results:**

We found that 63% of the studies reported daily time spent in SB and in MVPA, but fewer reported LPA, moderate PA, and vigorous PA. The average time spent in SB was 553.34 min/day, in LPA was 299.77 min/day, and in MVPA was 33.87 min/day. In occupational subgroup analysis, we observed that office workers had 2.3 h more SB, 2.4 less hours LPA, and 14 min less MVPA per day than nurses. However, most studies either did not specify workers’ occupations or grouped occupations. Shift work and workplace facilities significantly influenced SB and PA, but organizational factors affecting these behaviors were not sufficiently investigated (e.g., occupation type, work environment and workplace facilities, and shift work).

**Conclusions:**

More research is needed to explore SB and PA patterns within occupational subgroups. Additionally, it is important to explore work-related individual (e.g., job task), interpersonal (e.g., social support from colleagues), organizational (e.g., work policy), and environmental factors influencing SB and PA. Future studies should also investigate the association of these factors with SB and PA.

**Supplementary Information:**

The online version contains supplementary material available at 10.1186/s12889-024-19449-y.

## Introduction

Too much sedentary behavior (SB) and too little physical activity (PA) place adult workers at risk for chronic illness [1–3]. SB is defined as “any waking behavior characterized by an energy expenditure ≤ 1.5 metabolic equivalents (METs), while in a sitting, reclining or lying posture [4], and PA is defined as body movements facilitated by skeletal muscles that lead to energy expenditure [5]. High SB is positively associated with all-cause mortality, including death from cancer and other chronic disease [6], while PA provides health benefits to reduce mortality [7]. Nevertheless, 46% of U.S. adults aged 18 to 64 years did not meet aerobic-activity or muscle-strengthening PA guidelines for Americans in 2020 [8]. Also, a recent U.S. Centers for Disease Control and Prevention report showed that over 25% of Americans aged ≥ 18 years were physically inactive [9]. Because adult workers spend a significant amount of time working, work-related patterns of SB and PA contribute significantly to total SB and PA [10, 11], but the relationship between work related SB and PA and total SB and PA is complex. People with physically active occupations can be highly sedentary during non-working hours, thereby offsetting the potential health benefits derived from the PA accumulated during working hours, the PA paradox [12]. Thus, the combined patterns of SB and PA must be understood to better manage workers’ health.

Sedentary work is on the rise, contributing to increased SB among workers and reduced overall PA levels [10, 13]. For occupational settings, there is currently no standard definition of sedentary work based on energy expenditure [14, 15]. In addition, SB and PA are often considered polar opposites [5], but this is not reflected by studies. For example, occupation types with the lowest reported SB are food preparation and sewing and farming, fishing, and forestry, but working groups showing the highest moderate to vigorous physical activity (MVPA) are healthcare support and community and social service [16]. Thus, it remains unclear which occupations and subgroups within occupations have the highest and lowest SB and PA, and how organizational factors affect these measures. To address this gap, our review focuses on studies that capture both measures.

Office work is generally classified as sedentary [13, 16] and is considered a high-SB occupation; however, the occupational groups with the highest PA levels have varied in different studies [13, 16]. In addition, only a few organizational factors that influence SB and PA have been identified, namely the work environment [17], benefits of social support in workplace [18], worksite culture [19], and worker characteristics. This lack of information makes it difficult to understand whether workers’ lifestyles, workplace behaviors, and environments are barriers or motivators for SB and PA.

Although previous studies have described SB and PA in adult workers, most of their results have been based on self-reported measures or on measurement of SB or PA but not both [11, 17]. Moreover, the accelerometry studies that have been conducted had challenges in terms of being representative of each occupation due to a small sample size [20]. Because estimates of SB and PA have differed significantly depending on whether they were self-reported or accelerometer-measured [21], we focused our study on SB and PA outcomes measured in larger samples by accelerometry only to maximize the accuracy of our findings. The aims of this review were as follows:

1. Summarize the evidence of daily SB and PA measured by accelerometry for different occupations in large-scale studies.
2. Identify organizational factors influencing SB and PA

## Methods

### Search strategy

The comprehensive literature search for this systematic review and meta-analysis followed Preferred Reporting Items for Systematic Reviews and Meta-Analyses (PRISMA 2020) guidelines [22]. Through April 25, 2024, six databases were searched for relevant literature: Cumulative Index of Nursing and Allied Health Literature (CINAHL) Complete EBSCO, Excerpta Medica Database (Embase), PubMed, Scopus, SPORTDiscus EBSCO, and Web of Science. Search terms were selected to focus on studies addressing exercise or SB and PA in working populations (see Appendix A). A reference librarian was consulted for the selection of databases and the development of search terms. In addition to the database search, we manually reviewed the reference lists of the included studies in an attempt to identify other relevant studies.

### Study selection and data extraction

The inclusion criteria were as follows: (a) primary research published in English in peer-reviewed journals, (b) studies specifically targeting the working population, and (c) sample sizes > 75, (d) objective measurement of both SB and PA for seven consecutive days using accelerometry following National Health and Nutrition Examination Survey PA monitoring guidelines [19, 20], (d) inclusion of at least 3 valid days of accelerometer data, and I reporting of time spent in SB and PA as means with standard deviations (SDs) or confidence intervals (CIs) to allow consistent comparison of outcome variables.

Because appropriate sample sizes for feasibility and pilot studies range from 10 to 75 [23], our review targeted studies with samples > 75 to obtain reliable and valid results that would adequately reflect variations across occupational groups. We chose the sample threshold of 75 to capture studies that potentially had more heterogeneous samples within occupational groups; this choice was based on Teresi et al.’s (2023) recommendation that sample sizes of 70 to 100 per group should be required for detection of group differences in pilot studies [24]. Also, although 4 valid days of accelerometer data are desirable [25], we set our inclusion criterion at 3 valid days to maximize the number of large-sample studies included [26].

We excluded studies of the general population when working status was only described as part of the demographic characteristics; measured only SB or PA because there is a known strong negative relationship between SB and LPA [27]; reported outcomes as percentages, METs, or step-counts; or reported outcomes as median and/or interquartile range.

**Fig. 1:**
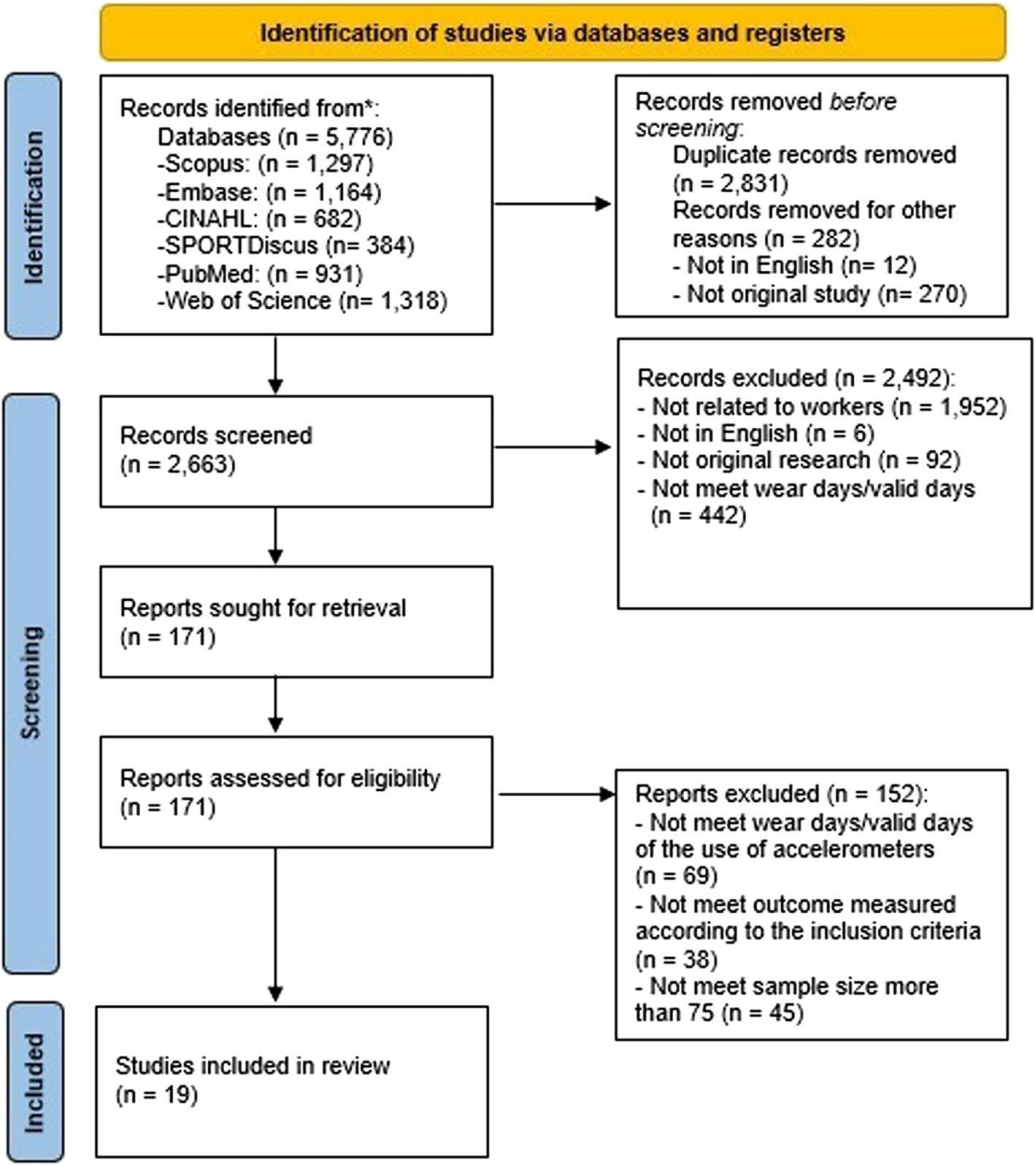
PRISMA 2020 Flow Diagram of Study Selection Process for Systematic Review

This review was prospectively registered with the PROSPERO database of systematic and meta-analysis reviews (CRD42022374343). The search yielded 682 articles in CINAHL, 1,164 in Embase, 931 in PubMed, 1,297 in Scopus, 384 in SPORTDiscus EBSCO, and 1,318 in Web of Science (Fig. 1). We removed 2,831 duplicates using EndNote 20 [28]. The first author screened titles and abstracts using EndNote, and 2,492 articles were excluded. The remaining 171 articles were independently examined by three authors (SP, SL, KW) [29] and 19 articles met criteria for inclusion (see Fig. 1). We extracted publication information, sample characteristics, research design, measurements of the two main outcomes (i.e., total time/day in SB and in PA), and relevant findings (see Supplementary Table 1). Completeness and accuracy of the data extracted were assessed and double-checked by four authors (SP, SL, SW, KW). The corresponding/first authors of three articles were contacted for additional information, and two responded. All main outcomes were reported as min/day.

### Risk of bias in selected studies

**Fig. 2:**
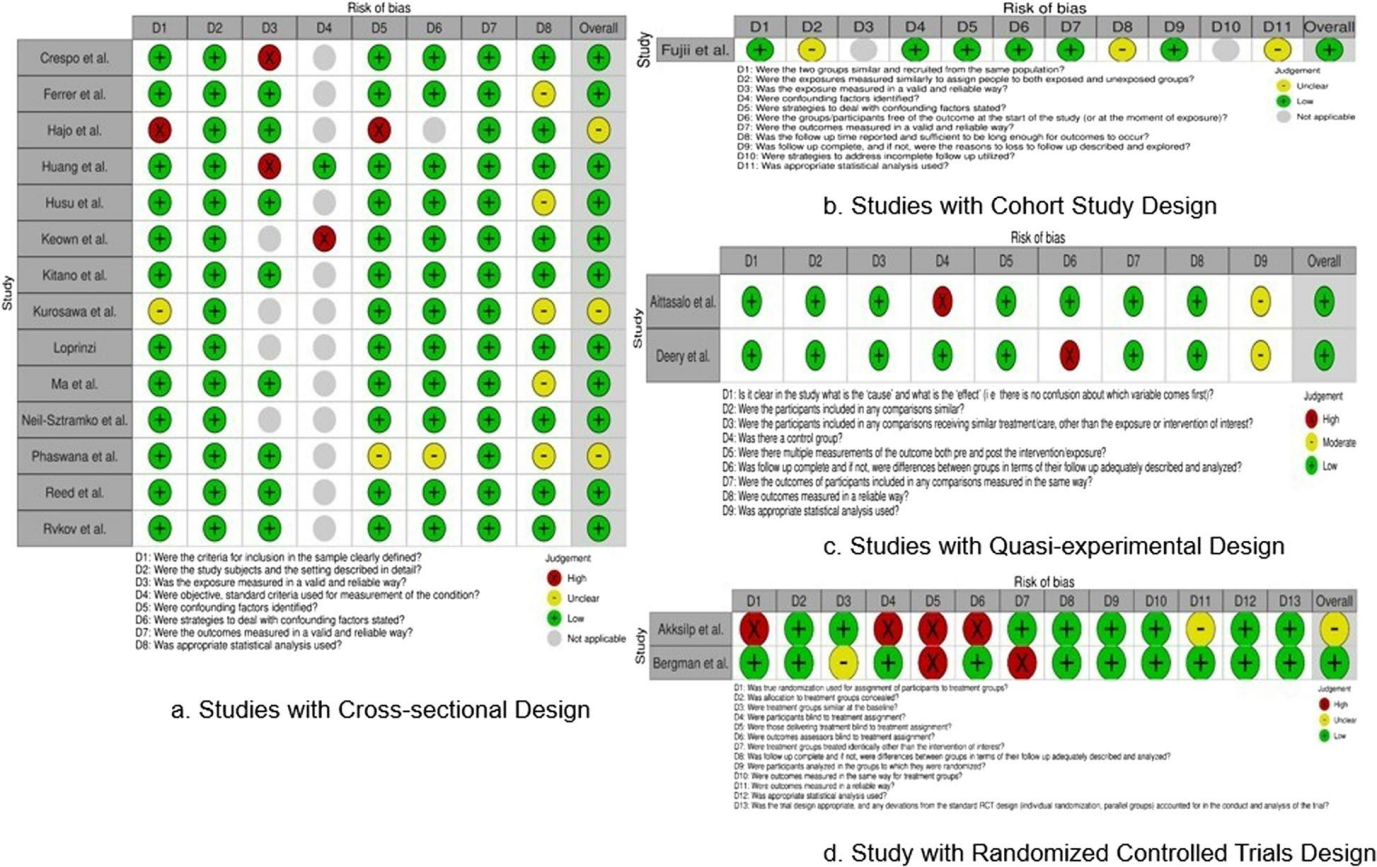
Risk of Bias Summary by JBI

The risk of bias in the selected studies was assessed using the Joanna Briggs Institute’s (JBI) critical appraisal tools. We used the appropriate JBI tool for each research design (see Fig. 2): cohort (11 questions) [30], cross-sectional (8 questions) [30], quasi-experimental (9 questions), and randomized controlled trial (13 questions) [32]. Each question was rated using four categories: “Yes,” “No,” “Unclear,” or “Not applicable.”

Two authors (SL and SW) independently assessed the quality of the selected studies and resolved any disagreements through discussion. If disagreements remained unresolved, a third author (SP) was consulted to reach a consensus. There is no established guideline for determining scoring values using the JBI tool [30–32]. Therefore, we calculated the proportion of “Yes” divided by the total number of questions excluding “Not applicable” in each study. We evaluated the degree of risk (i.e., low, moderate, and high risk of bias) [33, 34].

### Meta-analysis

For the meta-analysis, two authors (SP and KW) prepared a coding list of variables of interest based on Supplementary Table 1 (i.e., study number, publication year, sample size, occupation, SB, and PA levels). Given that most study designs were cross-sectional, we selected the baseline outcome values for studies with multiple outcomes at various time points [35, 36]. After double-checking the variables for consistency, a senior biostatistician (PV) verified the list [37]. Ten studies examined time spent in both SB and PA across the total study sample using a combination of working days and non-working days [38–47]. For studies reporting multiple outcomes at various time points, we selected the baseline outcome values for consideration. [45, 47]. Seven of the 10 studies were included in the meta-analysis; they all used hip/waist-mounted Actigraph accelerometers. The other three were excluded because one reported data from a wrist-worn device that produces higher estimates of PA [44] and the others used an accelerometer (Actical [48] and AX3 [46]) that does not produce data comparable to Actigraph data [49, 50].

We used﻿ Stata version 18.0 for the meta-analysis [51], employing the mean values and standard errors (SEs) for daily SB and daily PA. SDs and CIs were converted into SEs [52]. Based on Cochrane’s guideline [52], SDs were calculated using the formula SE$\times \sqrt{sample size}$, and CIs were calculated using the formula SD$= \sqrt{sample size} \times (upper limit-lower limit)/ 3.92$ (given the 95% CI range). To calculate the pooled SD for two groups [45, 47], we used the formula [53] SD_pooled_$=\sqrt{\frac{\left(n1-1\right)\left(SD1\right)({\text{SD}}1)+\left(n2-1\right)\left(SD2\right)(SD2)}{n1+n2-2}}$. A random-effects model was applied for the analysis due to expected variations across occupational groups in the meta-analysis [54].

## Results

The research purpose, design, sample size and characteristics, measures, main outcomes (i.e., time spent in daily SB and daily PA [min/day]), and other relevant findings for each selected study are summarized in Supplementary Table 1. Of the 19 selected studies, 14 were cross-sectional, two were quasi-experimental, two were randomized controlled trials (RCTs), and one was a cohort study. The sample size varied from 78 to 3,513 workers, and mean ages ranged from the mid-30s to early 50s. Four studies were conducted in Japan, and three studies each in the USA, and Canada. Two studies were conducted in Finland and one study each in Sweden, New Zealand, the UK, Thailand, Taiwan, South Africa, and Singapore. During our review, we classified the occupation types reported in the studies into four groups: (1) office workers [36, 41, 42, 45–47, 55], (2) occupation not specified (typically referred to only as “worker/employee”) [38, 48, 56–59], (3) grouped occupations (combinations of several occupations such as workers in banking services and at amusement parks) [35, 39, 44, 60], and (4) nurses [40, 43]. These classifications are further discussed under *“Organizational Factors Related to SB and PA”* below.

Most of the 19 studies reviewed were conducted prior to the COVID-19 pandemic, but data were collected for three studies [45, 46, 60] during the pandemic. Among these three studies [45, 46, 60], only Fujii et al.’s study compared SB and PA before and during COVID-19; they reported that office workers showed increased SB and reduced PA both on weekdays and weekends after the pandemic compared to before it [60].

Regarding measurement devices for SB and PA, 10 studies used the ActiGraph (53%); four [55, 56, 58, 60] used the Active style Pro HJA; and one each used the Fitbit [44], Actical [48], AX3 [46], Hookie AM [35], and UKK RM42 [59]. Two studies [36, 42] that applied the ActiGraph also used the activPAL to assess SB in greater detail. The device wear location was most commonly the waist or hip (79%; 15 of 19 studies); three [44, 46, 57] studies employed a wrist-worn device, and one [56] did not identify the wear location. Twelve studies [35, 36, 38, 39, 41, 42, 46–48, 56, 57, 59] reported 7 consecutive days of monitoring. Of 19 studies, the number of required valid days of data varied: ≥ 4 days in 10 studies [38, 40, 41, 43, 46–48, 57–59], ≥ 3 days in one study [39], and 14 days in one study [44]. Additional seven studies included data for a mix of working and weekend days such as 3 working days and 1 non-working day [36, 56], 2 working days and 1 non-working day [42, 55, 60], and 3 working days only [35, 45]. Most studies reported using 60-s epochs [36, 38, 39, 41, 42, 44, 45, 47, 48, 55, 57, 60].

No studies showed a high risk of bias. Most studies had a low risk (16 of 19 studies) [35, 36, 38, 39, 41–44, 46–48, 55, 57–60], and three [40, 45, 56] had a moderate risk of bias (see Fig. 2– Figs. 2a-d). Two of the studies [40, 56] with a moderate risk of bias were cross-sectional and raised concerns related to a lack of clear selection criteria. In addition, one RCT study [45] posed concerns regarding the blind assignment of treatment; it was unclear how blinding could have been maintained for each participant and researcher and how to avoid cross-contamination between control and intervention groups.

### Profiles of total daily SB and PA time

Both daily SB and MVPA during waking hours were reported in 12 (63%) of the 19 studies [38–48, 57]. For profile calculations, we excluded Hajo et al.’s study [40] because it employed the same dataset as was used in Reed et al.’s study [40]. Across seven of these studies employing a waist-worn ActiGraph and a total of 3,176 workers [38, 39, 41–43, 45, 47], the mean SB time was 553.34 min/day (SD 91.54 min/day), and the mean MVPA time was 33.87 min/day (SD 21.68 min/day) [38, 39, 41–43, 47]. Also, our review revealed that relatively low proportions of studies reported LPA, moderate PA, and vigorous PA: only 42% [40–46, 57] reported LPA, and 16% [40, 43, 44] reported both moderate PA and vigorous PA. Across four studies with waist-worn ActiGraph and a total of 957 workers, the mean LPA was 299.77 min/day (SD 74.96 min/day) [41–43, 45].

The main outcomes, time in daily SB and PA, were not consistently reported (Supplementary Table 1). Studies reported results based on commute mode [39], work shifts [48, 57], or sample characteristics [36, 41, 47, 59], such as sex.

### SB and PA by specific timeframe

Eight of 19 studies reported SB and PA according to specific timeframes such as working days, working hours during working days, and non-working days (see Supplementary Table 2) [35, 36, 42, 45, 55, 56, 58, 60], but results were inconsistently reported across those studies. Only one [42] reported detailed outcomes for all three categories: working days (also addressing working vs non-working hours), non-working days, and mixed days (encompassing both working and non-working days). An additional seven studies addressed only one or two of these categories in their outcomes (see Supplementary Table 2).

During our review, we observed no consistent trends in SB or PA intensity between working days and non-working days across studies. For example, in two studies [36, 42], detailed SB and PA outcomes for a total of 158 workers were obtained using both the activPAL (for SB) and ActiGraph (for PA). In those studies [36, 42], workers showed more SB and less PA on working days. Specifically, they had 49 min/day more SB, 29 min/day less LPA, and 3.34 min/day less MVPA on working days than on non-working days [36, 42]. However, different outcomes were reported by three studies [55, 56, 58] using an HJA device; those studies involved totals of 629 workers on working days [56, 58] and 1,663 workers on non-working days [55, 56]. Across these studies [55, 56, 58], workers had 1,019.5 min/day less SB on working days but were more physically active on non-working days, showing 149.95 min/day more LPA and 19.3 min/day more MVPA than on working days.

### Meta-analysis of SB and PA

**Fig. 3:**
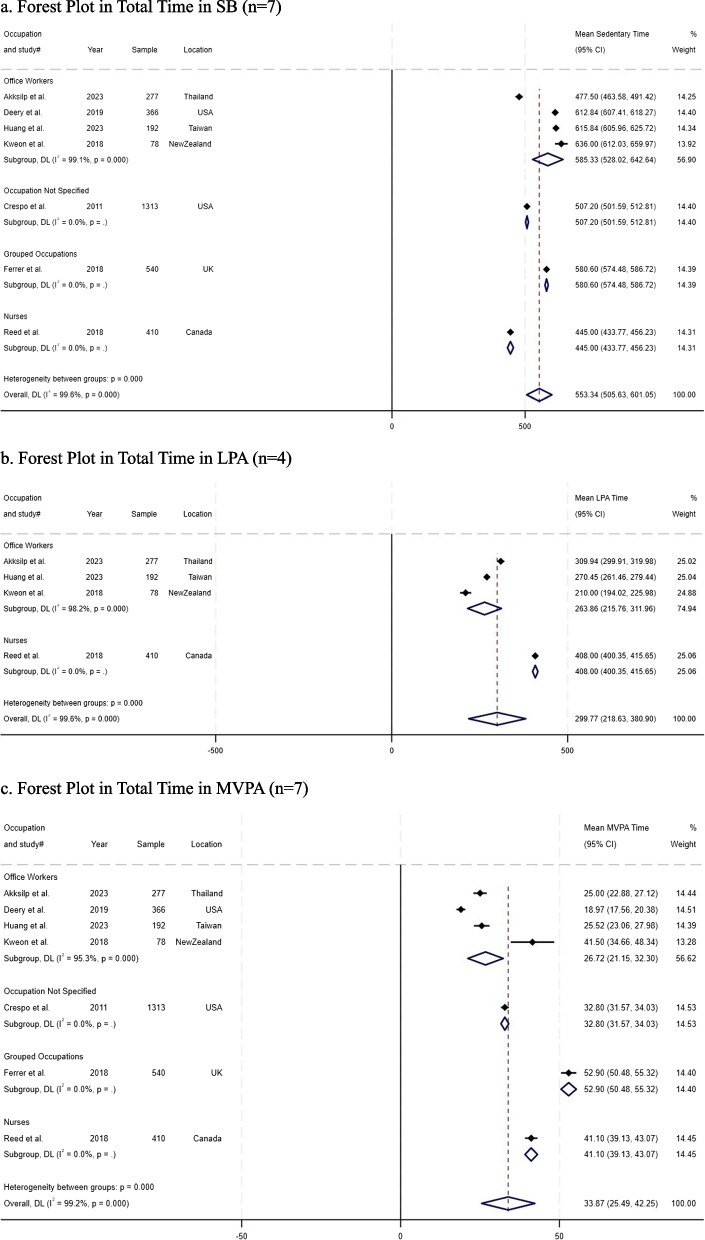
Forest Plots in Total Time in SB, LPA, and MVPA. **a**. Forest Plot in Total Time in SB (*n* = 7). **b**. Forest Plot in Total Time in LPA (*n* = 4). **c**. Forest Plot in Total Time in MVPA (*n* = 7)

Seven studies with a total of 3,176 workers were included in the meta-analysis [38, 39, 41–43, 45, 47]. Occupation type was the only common factor among them, and so we conducted a subgroup analysis by occupation (see Fig. 3). Workers studied included nurses in one study (*n* = 410) [43], office workers in four studies (*n* = 913) [41, 42, 45, 47], workers with occupation not specified in one study (*n* = 1,313) [38], and workers with grouped occupations in one study (*n* = 540) [39].

The time spent in SB across all seven studies [38, 39, 41–43, 45, 47] averaged 553.34 min/day (95% CI 505.63 – 601.05., *p* < 0.001., Fig. 3-a). Based on subgroup analysis, nurses [43] had the lowest SB (mean = 445 min/day, 95% CI 433.77 – 456.22), followed by workers with occupation not specified [38] (mean = 507.20 min/day, 95% CI 501.59 – 512.81), and workers with grouped occupations [39] (mean = 580.60 min/day, 95% CI 574.48 – 586.72). Office workers [41, 42, 45, 47] had the highest SB (mean = 585.33 min/day, 95% CI 528.02 – 642.64., *p* < 0.001).

As shown in Fig. 3-b, the mean time spent in LPA was 299.77 min/day (95% CI 218.63 – 380.90., *p *< 0.001) [41–43, 45]. For subgroup analysis, only two occupations were used: Office workers [41, 42, 45] had lower LPA (mean = 263.86 min/day, 95% CI 215.76—311.96., *p* < 0.001) than nurses [43] (mean = 408 min/day, 95% CI 400.35—415.65). In addition to all workers, the average time spent in MVPA [38, 39, 41–43, 45, 47] was 33.87 min/day (95% CI 25.49 – 42.25., *p* < 0.001., see Fig. 3-c). Office workers had the lowest MVPA (mean = 26.72 min/day, 95% CI 21.15 – 32.30., *p* < 0.001), followed by those with occupation not specified (mean = 32.80 min/day, 95% CI 31.57 – 34.03) and nurses (mean = 41.10 min/day, 95% CI 39.13 – 43.07). Workers with grouped occupations had the highest MVPA (mean = 52.90 min/day, 95% CI 50.48 – 55.32). Compared to office workers (lowest MVPA), workers with grouped occupations (highest MVPA) had 26.18 min/day higher MVPA.

### Organizational factors related to SB and PA

Organizational factors influencing SB and PA were insufficiently addressed in the 19 studies, as the only organizational factor considered in every study was occupation type. The most common occupation type was office workers [36, 41, 42, 45–47, 55]. Other occupation types were nurses [40, 43], workers with occupations not specified [38, 48, 56–59], and workers with grouped occupations [35, 39, 44, 60].

The organizational factors most commonly found to influence SB and PA were on-site work environment and facilities [35, 38, 43, 47] and shift work [40, 43, 48, 57]. Regarding on-site work environment and facilities [35, 38, 43, 47], two studies [35, 47] involved interventions that adjusted the work environment to promote decreased SB and increased PA. For instance, Deery et al. [46] reported that PA calorie expenditure labels implemented in worksite cafeterias slightly reduced SB and increased PA [47]. As for shift work, shift work type [43, 48, 57], shift length [43], and shift work disorders [40] were reported as influencing SB and PA, and specific shift conditions influenced SB and PA differently. For example, rotating shift workers had less SB and more LPA than day shift workers [57]. In addition, other organizational factors influencing SB and PA were absenteeism [40], work performance efficiency [58], and commute mode and commute distance [39]. Although unit-peer support was included as one component of the intervention addressed by Aitassalo et al., [35] no significant influence of social support on SB or PA was reported in their study or in any others.

## Discussion

Having estimated daily SB and PA using accelerometry, our review indicates that adult workers average 9.22 h per day in SB, 5 h per day in LPA, and 0.56 h per day in MVPA. Also, our review revealed that the only common organizational factor influencing SB and PA was occupation type. Notably, we found that insufficient organizational factors were examined in terms of their influence on SB and PA.

Our review highlighted two specific occupations (nurses and office workers), suggesting that estimating SB and PA based on workers’ individual characteristics, such as occupation and age, helps capture these parameters more meaningfully than estimating them for working adults as a whole. Compared to a previous review [13] that included studies employing a combination of various devices to measure SB and PA and varied sample sizes of working adults, our waist-worn Actigraph-derived SB and PA estimates were based on larger samples. However, we obtained similar findings: a 12.46-min lower SB and a 9.53-min lower LPA, but a 19-min greater MVPA. In addition, we compared our outcomes with previous studies [64, 65] examining a large sample of the general adult population included in the U.S. National Health and Nutrition Examination Survey, which like our study employed waist-worn Actigraph data. In comparison with those studies [64, 65], we found that workers (in our review) had more SB, less LPA [64], and more MVPA than in Kim and Kang’s [64] study [65] but less MVPA than in Fishman et al.’s [63] study [64]. These differences in outcomes may be due to the age range of the sample. Previous studies [64, 65] included older adults aged 65 or more. Approximately 50% of the studies (10 of 19) either did not clearly classify occupations within groups or used workers whose occupations could not be differentiated when reporting SB and PA. Despite these limitations, our review highlighted findings for two specific occupations: office workers and nurses. For office workers, our results support previous studies’ [13, 16] findings that office workers had the highest SB. With respect to PA, however, we found that office workers showed the lowest MVPA, whereas a prior review [13] reported that office workers had more MVPA than other occupations. Notably, the term “office worker” was typically not defined in past studies [66–69]. To better understand the profiles of office workers and create tailored strategies to increase their PA and decrease their SB, subgroups of office workers need to be defined and classified. One way to do so is to use occupational codes such as the Standard Occupational Classification System (e.g., 43–0000 for Office and Administrative Support Occupations) [70] and/or the North American Industry Classification System (e.g., 561,110 for Office Administrative Services) [71]. To achieve greater consistency in research reporting, these occupational codes can be converted using “autocoder” software applications [72]. As for the nursing occupation, a previous study targeting nurses [73] has reported findings similar to ours. In that research, nurses showed lower SB (mean = 445 min/day) [73] and higher MVPA compared to other healthcare occupations. To better understand the facets of multiple occupations across industries, we recommend that future studies investigate specific occupations and groups of occupations and measure each outcome for different occupations within specific industries.

Moreover, our review did not find sufficient organizational factors that influence SB and PA, with our key findings being limited to the effects of shift work and onsite-work facilities on work performance and benefits to the company (e.g., work performance efficiency, absenteeism). In general, the reviewed studies neglected to examine social organizational factors such as social support and workplace climate, workplace benefits (e.g., a PA work wellness program), mental stress caused by the job, and individual organizational factors (e.g., job task and home office work environment). Nevertheless, we did find that a few studies reported on partial organizational factors such as workplace facilities. The dominant organizational factors that we identified as influencing SB and PA, namely occupation types, may not fully capture the evolving nature of how job tasks are impacted by technological advancement [74]. Therefore, it is essential that more studies explore diverse occupational changes (e.g., job characteristics [job task] and workplace [home-office]) and micro–macro level factors that influence SB and PA. Such studies may eventually enhance work climate and policy support, thereby reducing SB and increasing PA for workers.

Following the COVID-19 pandemic, workers have reported increased SB and reduced PA [75], with remote workers in particular having experienced a significant increase in SB and decrease in PA [76, 77]. However, those studies were based on self-reported data. Brusaca et al.’s [77] study, which used accelerometry, supported the finding that sitting time was higher in remote workers than in on-site workers [78]. There findings were also supported by our review: among the 19 studies we reviewed, Fujii et al.’s [59] study [60] reported that office workers had more SB and less PA after the pandemic than before. To mitigate these negative trends in SB and PA, a combination of home working environment modifications and behavior-changing strategies have been recommended. Indeed, these changes have proven effective in improving remote workers’ mental health, reducing their SB, and enhancing their work performance [79]. These outcomes reflect the importance of designing workplaces, be it remote or traditional, that promote worker health and well-being. They also highlight the growing need to facilitate healthy remote work environments and establish a supportive organizational culture [77]. On the whole, future research should comprehensively examine the impact of home and traditional work arrangements and their associated environments on SB and PA.

### Strengths and limitations

One of the strengths of this review is its emphasis on objectively measured SB and PA using accelerometry. It helps to overcome the inherent bias in self-reported SB and PA, overestimation of PA and under-estimation of SB [21]. In addition, our meta-analysis compared SB and PA in four working groups (i.e., workers with grouped occupations, nurses, office workers, and workers with occupation not specified), thereby supporting results from the systematic review and enhancing the rigor and validity of our findings.

The study findings are subject to a number of limitations. First, due to our stringent inclusion and exclusion criteria, our study could not capture SB and PA across the range of occupations. For example, in the meta-analysis, we could compare only two identifiable occupation types—office workers and nurses. For studies that aggregated data and grouped results for multiple groups, we were unable to separate results by occupation. However, we believe that our approach was justified in providing a synthesis of the most reliable data available. Second, although most of the reviewed studies showed a low risk of bias, our findings regarding factors significantly influencing SB and PA should be interpreted with caution because several of the studies were cross-sectional, and we included those outcomes. Third, methodological differences among the reviewed studies made it challenging to synthesize their results. Studies used different devices; different device placements (waist/hip vs wrist); and different timeframes such as working days, non-working days, and a combination of the two. As an example of the issues arising from these differences, we did not include results based on data from wrist-worn devices [44, 57] because they have no standard cut points [80, 81]. To minimize outcome variances, our meta-analysis results for SB and PA are based solely on data generated by ActiGraph devices worn on the waist/hip [38, 39, 41–43]. Finally, because the aim of the review was to assess evidence regarding total daily SB and PA, we did not explicitly consider posture-specific accelerator measurements such as standing vs. sitting.

## Conclusion

Our review indicates that adult workers average 9.22 h per day in SB, 5 h per day in LPA, and 0.56 h per day in MVPA. Our review supports earlier reports of office workers having higher SB and lower PA than other groups of workers. Nurses had the lowest SB and highest PA. To better detect occupational subgroup differences using accelerometry, we suggest that future studies specify outcomes from grouped occupations and/or specific occupations from different industry categories.

There is limited evidence identifying organizational factors that influence SB and PA along with SB and PA outcomes. To more comprehensively understand SB and PA in working adults, it is essential to explore work-related individual (e.g., job task), interpersonal (e.g., social support from colleagues), organizational (e.g., work policy and work culture), and environmental factors (e.g., office facilities) influencing SB and PA and their associations with SB and PA outcomes. Specifically, we suggest that future studies investigate the impact of redesigning workplaces, as social support and interaction at work have been shown to influence SB and PA.

### Supplementary Information

Supplementary Material 1.

Supplementary Material 2.

## Data Availability

No datasets were generated or analysed during the current study.

## Abbreviations

CI: Confidential intervals
CINAHL: Cumulative index of nursing and allied health literature complete EBSCO
Embase: The excerpta medica database
JBI: Joanna Briggs Institute
LPA: Light physical activity
METs: Metabolic equivalents
MVPA: Moderate to vigorous physical activity
PA: Physical activity
PRISMA: Preferred reporting items for systematic reviews and meta-analyses
RCT: Randomized controlled trial
SB: Sedentary behavior
SD: Standard deviation
SE: Standard error
