# Supplementary material for: Sedentary behaviors and physical activity of the working population measured by accelerometry: a systematic review and meta-analysis
Source: BMC Public Health. 2024 Aug 6;24:2123. doi: 10.1186/s12889-024-19449-y (PMC11302194; doi:10.1186/s12889-024-19449-y)
Supplement: Supplementary file 1 — Supplementary Material 1. [file 12889_2024_19449_MOESM1_ESM.pdf]

Appendix A. Search Terminology That Used in Each Database

The Cumulative Index of Nursing and Allied Health Literature (CINAHL) Complete EBSCO:

1.

(((MH "Posture") OR (MH "Exercise+") OR (MH "Locomotion+") OR (MH "Physical Activity") OR (MH "Sports+") OR "Physical activity" OR "Physical activities" OR Exercis* OR walk* OR "activity pattern" OR "activity level" OR "step count" OR stepcount* OR sport* OR "Physical behaviour" OR "Physical behavior" OR running) AND ((MH "Sitting") OR (MH "Life Style, Sedentary") OR (MH "Supine Position") OR sedentary OR "Physical Inactivity" OR Sitting OR posture OR sit OR sat OR seat* OR lying))

2.

(MH "Accelerometry+") OR (MH "Fitness Trackers") OR (MH "Actigraphy") OR (MH "Pedometers") OR (MH "Accelerometers") OR Accelerometry OR accelerometer OR accelerometers OR Actigraphy OR actigraph OR actigraphs OR activpal OR Actical OR sensecam OR inclinometer OR inclinometers OR inclinometric OR inclinometry OR pedometer*

3.

(MH "Occupations and Professions+") OR (MH "Named Groups by Occupation+") OR (MH "Health Occupations+") OR (MH "Occupational Safety") OR (MH "Occupational Medicine") OR (MH "Occupational Justice") OR (MH "Occupational Health Services+") OR (MH "Occupational Health Nursing") OR (MH "Occupational Health") OR (MH "Employment+") OR (MH "Work Environment+") OR Workplace* OR Work-Site* OR Worksite* OR Work OR Job OR Employee* OR occupation OR Worker* OR company* OR offic* OR busines*

1 AND 2 AND 3

The Excerpta Medica Database (Embase):

('exercise'/exp OR 'physical activity'/exp OR 'motor activity'/de OR ("Physical activity" OR "Physical activities" OR Exercis* OR walk* OR "activity pattern" OR "activity level" OR "step count" OR stepcount* OR sport* OR "Physical behaviour" OR "Physical behavior" OR running):ti,ab) AND ('sitting'/exp OR 'reclining position'/exp OR 'sedentary lifestyle'/exp OR 'physical inactivity'/exp OR (sedentary OR "Physical Inactivity" OR Sitting OR postureOR sit OR sat OR seat* OR lying):ti,ab) AND ('activity tracker'/exp OR 'accelerometry'/exp OR 'actigraph'/de OR 'actimetry'/exp OR (Accelerometry OR accelerometer OR accelerometers OR Actigraphy OR actigraph OR actigraphs OR activpal OR Actical OR sensecam OR inclinometer OR inclinometers OR inclinometric OR inclinometry OR pedometer*):ti,ab) AND ('named groups by occupation'/exp OR 'occupational'/exp OR 'occupation and occupation related phenomena'/exp OR 'occupation'/exp OR 'work'/exp OR 'occupational health'/exp OR 'occupational health service'/exp OR 'work environment'/exp OR (Workplace* OR Work-Site*

OR Worksite* OR Work OR Job OR Employee* OR occupation OR Worker* OR company* OR offic* OR busines*):ti,ab)

PubMed:

1.
("Exercise"[Mesh] OR "Motor Activity"[Mesh] OR "Walking"[Mesh] OR "Sports"[Mesh] OR "Physical activit*"[tw] OR Exercis*[tw] OR walk*[tw] OR "activity pattern*"[tw] OR "activity level*"[tw] OR "step count*"[tw] OR stepcount*[tw] OR sport*[tw] OR "Physical behaviour*"[tw] OR "Physical behavior*"[tw] OR running[tw]) **AND** ("Sitting Position"[Mesh] OR "Posture"[Mesh] OR "Sedentary Behavior"[Mesh] OR sedentary[tw] OR "Physical Inactivity"[tw] OR Sitting[tw] OR posture[tw] OR sit[tw] OR sat[tw] OR seat*[tw] OR lying[tw])
2.
"Fitness Trackers"[Mesh] OR "Accelerometry"[Mesh] OR Accelerometry[tw] OR accelerometer[tw] OR accelerometers[tw] OR "Actigraphy"[Mesh] OR Actigraphy[tw] OR actigraph[tw] OR actigraphs[tw] OR activpal[tw] OR Actical[tw] OR sensecam[tw] OR inclinometer[tw] OR inclinometers[tw] OR inclinometric[tw] OR inclinometry[tw] OR pedometer*[tw]
3.
"Occupational Groups"[Mesh] OR "Job Description"[Mesh] OR "Occupations"[Mesh] OR "Occupational Health"[Mesh] OR "Occupational Health Services"[Mesh] OR "Occupational Medicine"[Mesh] OR "Occupational Injuries"[Mesh] OR "Occupational Health Nursing"[Mesh] OR "Workplace"[Mesh] OR Workplace*[tw] OR Work-Site*[tw] OR Worksite*[tw] OR Work[tw] OR Job[tw] OR Employee*[tw] OR occupation[tw] OR Worker*[tw] OR company*[tw] OR offic*[tw] OR busines* [tw]
1 AND 2 AND 3

Scopus:

TITLE-ABS-KEY((("Physical activity" OR "Physical activities" OR Exercis* OR walk* OR "activity pattern" OR "activity level" OR "step count" OR stepcount* OR sport* OR "Physical behaviour" OR "Physical behavior" OR running) AND (sedentary OR "Physical Inactivity" OR Sitting OR posture OR sit OR sat OR seat* OR lying)) AND (Accelerometry OR accelerometer OR accelerometers OR Actigraphy OR actigraph OR actigraphs OR activpal OR Actical OR sensecam OR inclinometer OR inclinometers OR inclinometric OR inclinometry OR pedometer*) AND (Workplace* OR Work-Site* OR Worksite* OR Work OR Job OR Employee* OR occupation* OR Worker* OR company* OR offic* OR busines*))

SPORTDiscus EBSCO:

1.

(("Physical activity" OR "Physical activities" OR Exercis* OR walk* OR "activity pattern" OR "activity level" OR "step count" OR stepcount* OR sport* OR "Physical behaviour" OR "Physical behavior" OR running) AND (sedentary OR "Physical Inactivity" OR Sitting OR posture OR sit OR sat OR seat* OR lying))

2.

Accelerometry OR accelerometer OR accelerometers OR Actigraphy OR actigraph OR actigraphs OR activpal OR Actical OR sensecam OR inclinometer OR inclinometers OR inclinometric OR inclinometry OR pedometer*

3.

Workplace* OR Work-Site* OR Worksite* OR Work OR Job OR Employee* OR occupation* OR Worker* OR company* OR offic* OR busines*

1 AND 2 AND 3

Web of Science:

TOPIC((("Physical activity" OR "Physical activities" OR Exercis* OR walk* OR "activity pattern" OR "activity level" OR "step count" OR stepcount* OR sport* OR "Physical behaviour" OR "Physical behavior" OR running) AND (sedentary OR "Physical Inactivity" OR Sitting OR posture OR sit OR sat OR seat* OR lying)) AND (Accelerometry OR accelerometer OR accelerometers OR Actigraphy OR actigraph OR actigraphs OR activpal OR Actical OR sensecam OR inclinometer OR inclinometers OR inclinometric OR inclinometry OR pedometer*) AND (Workplace* OR Work-Site* OR Worksite* OR Work OR Job OR Employee* OR occupation* OR Worker* OR company* OR offic* OR busines*))
