# Supplementary material for: Sedentary behaviors and physical activity of the working population measured by accelerometry: a systematic review and meta-analysis
Source: BMC Public Health. 2024 Aug 6;24:2123. doi: 10.1186/s12889-024-19449-y (PMC11302194; doi:10.1186/s12889-024-19449-y)
Supplement: Supplementary file 2 — Supplementary Material 2. [file 12889_2024_19449_MOESM2_ESM.docx]

**Supplementary Tables**

**Table 1**

*Summary of Selected Studies (n=19)*

| **Reference** | **Research**  **purpose** | **Finalized sample size for the analysis** | **Sample**  **characteristics** | **Research design/**  **Intervention**  **characteristics/ Theoretical framework** | **Assessment measurements on SB and PA** | **Other main measures^#^** | **Total SB (min./day)**  **M [SD or 95%CI]** | **Total PA (min./day)**  **M [SD]** | **Other relevant findings/ Notes** |
| --- | --- | --- | --- | --- | --- | --- | --- | --- | --- |
| Aittasalo (2017)^ | To evaluate the 1-year moving to business intervention effects including whether the intervention works small-medium level of workplaces | N= 266 for the baseline; N= 175 for the 1-year follow-up | Occupation:  Grouped occupations (workers at banking services, climate control, congress and concert center, regional marketing, media house, banking services, training institute, technology center, media house, amusement park, and theater)  Setting: small to medium-sized workplaces (up to 249 employees) in Finland  Age: M 42.6 (SD 10.9) years  Sex: 107 males, 189 females | Not identified (appeared to be a one-group pretest-posttest, quasi experimental design)  Each workplace adopted a tailored intervention based on mentoring. The intervention consisted of meetings, workshops, campaigns, and other supports (e.g., consultations and group exercise), implemented by the company's internal team members (i.e., management, human resources, working staff, and/or occupational health care). The internet-based platform to monitor PA and share it with other colleagues was offered to participants for free. The action plans were implemented by individual-working unit-organizational levels.  Socio-ecological model [Did not address how to apply in detail] | Device: a right side, hip-worn accelerometer (Hookie AM13)  Wear time: 7 consecutive days excluding water activities.  Valid days: at least 3 working days with the minimum of 10 h wear-time, and 4 hours of wear-time at work.  Epochs: a 6-sec epochs  Algorithm:  - CPM: Henri Vähä-Ypyä et al.’s (2015) cut-points  - Non-wear time: no movement detected in any epoch for at least 30 min | Work, Work ability,  Work engagement and recovery. | - | Baseline:  M= 237.3 [SD 80.7]  Follow up:  M= 236.0 [SD 76.1] | Baseline data showed participants had 24 SB breaks.  Changes in PA After the intervention:  1. During leisure time: total daily PA (M= 11 min), LPA (M= 8.1 min) and MVPA (M= 2.7) were decreased compared to baseline.  2. At work: total daily PA (M= 33.7 min), LPA (M= 30.9), and MVPA (M= 4.1) were increased while daily SB (M= 44.9 min) and SB breaks (M= 0.2) were decreased.  There were no significant differences in the changes of PA and SB according by tailored intervention constructs implemented in the workplaces.  Not reported for work and worker engagement and recovery outcome. |
| Akksilp (2023)^ | To assess the effect PA intervention to reduce SB | N=277 for the baseline, 247 for the 6month follow up  At baseline: 138 intervention group, 139 control group.  At 6month:  122 intervention group, 125 control group | Occupation: Officer workers under the Ministry of Public Health  Setting: Thailand office workers  Age: M= 38.6 (SD 10.4) years  Sex at baseline:  112 females, 28 males in intervention group, 116 females and 26 males in control group. | Two arm parallel cluster RCT  The 6month- intervention group was offered pedometer, social support (i.e., team movement break and alarm reminder at four times, team incentive), leadership support (e.g., sending a message from director), and environment encouraging PA (i.e., provided health informatic posters).  Socio-ecological model. | Device: a right side, waist-worn accelerometer (Atcigraph^TM^).  Wear time: 24 hours a day for 10 days during data collection.  Valid days: at least 3 days of working days.  Epochs: a 60-sec epochs for Actigraph.  Algorithm:  - CPM: Sasaki et al. (2011): SB<151, LPA 151-2689, Moderate PA 2690-6167, and vigorous PA >6167.  - Non-wear time: Choi et al.’s (2011) algorithm. | Individual PA: Fitbit smartwatch to track for real-time feedback (e.g., step counts, calories),self-reported PA questionnaire, Work productivity, and Musculoskeletal health | [Intervention]  Baseline:  M= 481 [SD 110]  Intervention: M=474 [SD 125]  [Control]  Baseline:  M= 492 [SD 107]  Intervention:  M= 496  [SD 114] | LPA:  [Intervention]  Baseline:  M= 323 [SD 84.9]  Intervention:  M= 317  [SD 94.6]  [Control]  baseline:  M= 320 [SD 85.6]  Intervention:  M= 317 [SD 88.4]  MVPA:  [Intervention]  Baseline:  M= 24.8 [SD 15.9]  Intervention:  M= 29.5  [SD 20.4]  [Control]  baseline:  M= 25.2 [SD 19.9]  Intervention:  M= 24.4 [SD 19.3] | The 6-month intervention did not have a significant effect on reducing sedentary behavior among Thai office workers.  Although there was not significant differences in groups, intervention group reduced SB and increased MVPA at 6month’ follow-up. |
| Bergman (2018)^ | To assess the effect of the use of a treadmill workstation at the office longitudinally | N= 80, who were either overweight or obese  40 intervention group, 40 control group | Occupation: Office worker at private company, government, and municipality or county  Setting: Swedish offices  Age:  -range: 40–67 years  Sex: 18 males, 22 females in both intervention and control group. | RCT  The intervention group was offered a treadmill workstation and received four emails, including information related to the negative effects due to SB and reminders to promote the use of the treadmill. | Devices: 1) a thigh worn, activPAL 3 or activPAL 3 micro-activity monitor, and 2) a waist worn, Actigraph wGT3x-BT    Wear time: 24 hours a day for 7 consecutive days for activPAL; 14 consecutive days during waking hours for Actigraph. They were measured at baseline, 2-, 6-, 10-, and 13-months.  Valid days: 1) activPAL: at least 10 h of wear time, at least 500 steps, and 95% or less of the time awake in sitting or standing. For work time: at least 4 h of wear time,18 at least 250 steps, and 95% or less of the time spent in sitting or standing, and 2) Actigraph: at least 10hours of wear time, 4 valid days (3 work days and 1 non-work day)  Epochs: a 60-sec epochs for Actigraph  Algorithm:  - CPM: a modified version of Freedson Adult VM3 (2011): LPA 201-2689; MVPA $\geq$2690  - Non-wear time: a modified version of the Choi algorithm, with 60 minutes of consecutive zero counts, no spike  tolerance, and a small window length of 1 minute as a definition of non-wear time by Migueles et al.’s (2017) study, using vector magnitude. | - | - | - | No total SB and PA (time) reported; see Table 2.  Daily walking time at weekdays increased 18 min between  baseline and 13 months in the intervention group and 1 min in the control group.  Daily LPA at weekdays increased between baseline and 13 months.  At work, intervention was effective in decreasing SB (standing M= 206 min at baseline and M= 191 min at 13 months; sitting M= 275 min and M= 271 min). |
| Crespo (2011) | To assess relationship between worksite PA policy and strategies, PA, and SB | N=1313 | Occupation: Occupation not specified  Setting: Maryland and Washington, D.C. regions, USA  Age: M= 45.18 (SD 10.18) years  Sex: 735 males, 577 females: 1 missing | A cross-sectional study | Device: a right hip worn, Actigraph    Wear time: 7 consecutive days during waking hours (except for water activities)  Valid days: ≥ 4days (10 valid hours which defined having ≤ 30 consecutive min of zero counts)  Epochs: a 60-sec epochs  Algorithm:  - CPM: Freedson et al.’s (1998): SB <101, MVPA ≥ 1952  - Non-wear time: having >30 consecutive minutes of zero counts. | International physical activity questionnaire, and a worksite PA promotion index summed by the modified version of the checklist of health promotion environments at worksites. | M 507.2 [SD 103.7] | MVPA:  M 32.8 [SD 22.8] | The worksite PA promotion index was associated with age.  The higher index was significantly correlated to greater total SB, MVPA, and total recreational PA while associated with reduction of PA at work.  Those meeting PA guidelines had a higher worksite promotion index.  Associated with higher total sedentary time: male gender, higher education, greater wear time.  Associated with higher total MVPA: younger age, male gender, higher education, greater wear time.  Non-whites had lower total MVPA. |
| Deery (2019) | To examine about how PA calorie-expenditure food labels effects to changes in PA | N= 366  144 intervention group, 222  control group | Occupation: Office workers (insurance company workers)  Setting: three Blue Cross and Blue Shield of North Carolina worksite cafeteria, USA  Age: M 42.2 (SD 10.2) years  -intervention: M 40.9 (SD 9.6) years  -control: M 43.0 (SD 10.5) years  Sex: 22.4% Males/ 77.6% Females | Two-group interrupted time series cohort study design: quasi- experimental design  One-year intervention; one place provided PA calorie-expenditure food labels at meals and two places provided calorie only labels: three places used the same products and recipes of meals. | Device: a right hip worn, Actigraph wGT3X-BT, worn during the  same months in the baseline year and intervention year.    Wear time: 7days in each year  Valid days: 8 h per day, 4days  Epochs: a 60-sec epochs (from authors)  Algorithm:  - CPM: Troiano et al.’s (2008)  - Non-wear time: Choi et al.’s (2011) algorithm with logs and visual inspection | The modified version of the community healthy activities model program for seniors PA questionnaire at baseline and at intervention | [Intervention]  Baseline:  M= 620.7 [SD 49.5]  Intervention: M= 620 [SD 55.9]  [Control]  Baseline:  M= 607.7 [SD 54.9]  Intervention:  M= 614.8  [SD 44.6] | MVPA:  [Intervention]  Baseline:  M= 18.0 [SD 12.6]  Intervention:  M= 18.7  [SD 14.6]  [Control]  baseline:  M= 19.6 [SD 14.3]  Intervention:  M= 19.2 [SD 13.7] | Intervention reduced SB (% of differences between two time: -0.1) and increased MVPA (% of differences between two time: 3.7) meanwhile control group showed increased SB and reduced MVPA. |
| Ferrer (2018) | 1) To investigate travel mode and PA for employees  2) To investigate the relationship between travel mode, and range of characteristics in multi-level. | N= 540 of 654 workers, who completed objectively PA measured | Occupation: Grouped occupations  (public administration, professional and  scientific organizations, retail, services, and manufacturing)  Setting: workplaces in urban areas, UK  Age: not addressed about M or range  Sex: 283 males, 371 females among 654 employees | A cross-sectional study: analysis of the baseline data from the Travel to work multi-center cluster RCT. | Device: a waist worn, Actigraph GT3X+    Wear time: 7 days during waking hours  Valid days: at least 3 days of at least 600 minutes duration  Epochs: a 10-sec epochs  Algorithm:  - CPM: Freedson et al.’s (1998): SB<100; MVPA≥1952  - Non-wear time: continuous periods of 60 minutes or more of zero | Global positioning system receivers, travel diaries,  and questionnaires including factors relating to car use and perceptions of the commute | M= 580.6 [SD 72.6]  · By travel mode:  -car:  M= 587.6  [SD 69.5]  -walks:  M= 568.1  [SD 62.2]  -public transportation:  M= 585.8  [SD 65.2] | MVPA:  M= 52.9 [SD 28.7]  · By travel mode:  -car:  M= 46.3 [SD 20.6]  -walks: M= 71.3 [SD 21.3]  -public transportation:  M= 59.5 [SD 26.6] | 11.1% of participants met PA guidelines  Walkers and public transport users accumulated more MVPA and were more active during the commute (compared to car users)  Participants who walked at least ten minutes during their commute had more possibility to have a shorter distance in commuting and a job related to SB.  Participants commuted by car had the highest SB [M= 587.6 (SD 69.5)] the most. Participants commuted by walking had the highest PA [M=71.3 (SD 21.3)].  No car access, and absence of free car parking lot in the workplace were associated to walking to work and use the transportation in their commute. |
| Fujii (2023)^ | To assess the changes in SB and PA from before to after COVID-19 | N= 536 | Occupation: Grouped occupations (Office workers or sales/service workers)  Setting: Japan  Age: M 53.3 (SD 8.9) years  Sex:  163 Male,  373 Female | 1-year longitudinal, a prospective cohort study. | Device: a hip worn, Active style Pro HJA 750-C  Wear time: at least 10 days awaking time excluding water-related activities and contact sports.  Valid days: ≥ 10 hours per day and ≥ 3 days of wear-time (≥ 2 weekdays and ≥1 weekends)  Epochs: a 60-sec epochs  Algorithm  - Cut points by MET: SB≤1.5 METs, LPA 1.6-2.9 METs, MVPA ≥3 METs  - Non-wear time: ≥ 60 min of consecutive zeros for counts. |  | - | - | After COVID-19, workers’ SB increased and PA decreased by about 10 min per day compared to prior to COVID-19. |
| Hajo (2020) | To determine whether the combination of sleep, MVPA, and sedentary time are associated with absenteeism, mood state, and shift work disorder in nurses | N = 342 | Occupation: Nurses  Setting: Hospitals in the Champlain Local Health Integration Network of Ontario, Canada  Age: M 43.1 (SD 11.8) years  Sex:  19 Male,  323 Female | Secondary analysis of data from the cross-sectional Champlain Nurses’ Study | Device: a right hip worn, ActiGraph GT3X  Wear time: 9 days excluding water-related activities  Valid days: ≥ 10 hours per day and ≥ 4 days of wear-time (maximum 7 days of data was used)  Epochs: a 15-sec epochs  Algorithm  - CPM: Freedson Adult VM3 (2011): SB ≤ 150; PA ≥ 2691  - Non-wear time: ≥ 60 min of consecutive zeros for counts with an allowance of up to 2 minutes of counts ranged 0- 150 | Daily activity log over 24 h, shift work disorder screening questionnaire, a short-form health and work performance questionnaire during a 4-week period, profile of mood states questionnaire | M= 443.7 [SD 111.2] | LPA: M= 409.6 [SD 78.5]  Moderate PA:  M= 38.2 [SD 18.5]  Vigorous PA:  M= 3.4 [SD 5.5]  MVPA: M= 41.5 [SD 20.6] | Authors categorized four groups depending on the results of combinations of sleep, MVPA, and SB  Absenteeism did not different among groups statistically significantly.  The four behavioral groups were significantly associated with mood disturbance (p=.01), and vigor subscale (p = .003) and shift work disorder (p = .043).  Participants meeting the MVPA guidelines were significantly associated with lower mood disturbance, lower anger, higher vigor, and lower fatigue, compared whom did not.  Low sedentary time was significantly associated with lower mood disturbance, higher vigor, and lower fatigue, compared to higher sedentary time.  SB time was significantly, negatively associated with sleep and MVPA in bouts. |
| Huang (2022) | To examine whether sleep regularity is associated with objectively measured SB and PA in full-time workers | N = 192 | Occupation: Office workers with full-time jobs during the daytime  Setting: Companies and organizations in Taiwan  Age: M 38.56 (SD 8.89) years  Sex:  54 Male, 138 Female | A cross-sectional study | Device: a hip-worn, ActiGraph wGT3x-BT  Wear time: 7 consecutive days as many  hours per day as possible, up to 24 hours per day  Valid days: ≥ 600 minutes of wearing time in a 24-hour day, excluding the nighttime sleep periods and non-wear time; ≥ 4 valid days; and/or ≥ 5 nights of sleep record  Epochs: a 60-sec epochs  Algorithm  - CPM: SB ≤ 99;  LPA 100-2019; MVPA: ≥ 2020  - Non-wear time: a consecutive period of zero counts for ≥ 60 min | Physical health status  Time records (daily sleep time, work start and stop times on workdays, and times they forgot or were unable to wear the accelerometers) | M= 615.84 [SD 69.84]  · By sex:  -men:  M= 616.44 [SD 77.58]  -women: M= 615.61 [SD 66.86] | 1) LPA: M= 270.45  [SD 63.59]  2) MVPA: M= 25.52 [SD 17.41]  · By sex:  -men: 1) LPA: M= 274.56  [SD 65.82]  2) MVPA: M= 31.27 [SD 20.58]  -women: 1) LPA: M= 268.85  [SD 62.87]  2) MVPA: M= 23.27 [SD 15.50] | As one of sleep irregularity indicator, the greater social jet lag was correlated having more SB while having less LPA.  No evidence of relationships between other indicators of sleep regularity and SB/PA time. |
| Husu (2023) | To evaluate associations of PA, cardiorespiratory fitness, and work ability. | N= 1,255 | Occupation: Occupation not specified  Setting: Finland  Age: range: 20- 69 years  Sex: 509 Male, 746 Female | A cross-sectional study | Device: a right hip worn, UKK RM42 (100Hz sampling)  Wear time: 7 days during waking hours except for water-activities  Valid days: ≥ 4 days  Epochs: a 6-sec epochs  Algorithm  - Cut points by MET: SB≤ 1.5 METs; LPA 1.6-2.9 METs; and MVPA ≥ 3.0 METs  - Non-wear time: continuous quiescent time is longer  than 120 min. | Work ability index | M= 541 [SD not identified]  ·By sex:  [Male]  1)Lying: M=78 [SD 47],  2)Reclining: M= 313 [SD 84],  3)Sitting: M= 172 [SD 64]  [Female]  1)Lying: M=70 [SD 39],  2)Reclining: M= 285 [SD 84],  3)Sitting: M= 172 [SD 62] | LPA: M=231,  Moderate PA: M= 45,  Vigorous PA: M= 3  [All SD not identified]  ·By sex:  [Male]  LPA: M=227 [SD 72].  Moderate PA: M= 47 [SD 23],  Vigorous PA: M= 4 [SD 8]  [Female]  LPA: M= 234 [SD 76]  Moderate PA: M= 44 [SD 23]  Vigorous PA: M= 3 [SD 6] | More PA and better cardiorespiratory fitness were associated with a high work ability. |
| Keown (2018)^ | To describe the patterns of SB and PA within office-based employees | N = 78  Academic (n= 23); Professional (n= 36); Administration (n= 19) | Occupation: Office workers(office-based University employees (i.e., administration-, academic-, and professional staff))  Setting: University of Otago, New Zealand  Age: M= 45 years  - range: 25-63 years  Sex: 18 Male, 60 Female | [Not identified] appeared to be a cross-sectional study | Device: 1) a right hip worn, ActiGraph GT3X+, and 2) a right thigh worn, ActivPAL3  Wear time: 7 days during waking hours  Valid days: ≥ 10 hours of wear time per day and ≥ 3 days (including ≥ 2 workdays)  Epochs: a 60-sec epochs  Algorithm  - CPM: SB < 150; prolonged SB: < 150 with ≥ consecutive 30 min; LPA 150 – 1951; MVPA ≥ 1952  - Non-wear time: (from author) Their wearing time was defined by self-report, and confirmed by visual inspection of the data. Because their wearing time was 14.6 hours, they addressed 9.4 h as non-wear time. | Activity device wear time diary | [ActiGraph]  M= 636 [SD 108]  [activPAL]  Sitting/lying: M= 588 [SD 162] | [ActiGraph]  1) LPA:  M= 210 [SD 72]  2) MVPA: M= 41.5 [SD 30.8]  [activPAL]  1) Standing: M= 198 [SD 84]  2) Stepping: M= 96 [SD 42] | [ActiGraph data] Participants spent more sedentary time during a workday than a non-workday (p < .001) and during work hours than nonwork hours (p < .001). [ActivPAL data] Similarly, participants spent more time sitting/lying on a workday than a non-workday (p < .001) and during work hours than nonwork hours (p < .001).  Based on the ActivPAL data, adjusted for age, administration staff spends 43.2 minutes less time sitting than professional staff (p = 0.009) and 33.6 minutes less time sitting than professional staff (p = 0.032) during work hours, while the occupation was not associated with sedentary behavior during work hours (p = 0.088) in ActiGraph data.  Adjusted for age, administration staff had 1.2 fewer prolonged sedentary bouts compared to academic staff (p= .001), and 1.5 fewer prolonged sedentary bouts compared to professional staff (p < .001).  Associations of age and sedentary behavior at work had little effect regardless of statistical adjustment for occupation. |
| Kitano (2022)^ | To investigate the associations between objectively measured PA and SB for each three specific-time domain and cardiometabolic health indicators among office workers | N = 1258  Manager (n = 293); and general staff (n = 965) | Occupation: Office worker  Setting: Employees living in Tokyo, Japan  Age: M= 49.1 (SD 8.3) years  Sex: 510 Male, 748 Female | A cross-sectional study  Secondary analysis of data from the Meiji Yasuda LifeStyle study, a prospective study | Device: a right hip worn, Active style Pro HJA750-C  Wear time: ≥ 10 days during wake hours, except for water-based activities or contact sports  Valid days: ≥ 10 hours, with ≥ 2 valid work days and ≥ 1 valid non-work days  Epochs: a 60-sec epochs  Algorithm  - Cut points by MET: SB≤ 1.5 METs; LPA 1.6-2.9 METs; and MVPA ≥ 3.0 METs  - Non-wear time: 60 consecutive minutes with activity counts below the detection limit | Job title, employment status | - | - | PA and SB during non-working time on workdays were only significantly associated with cardiometabolic health -- 1) SB was related to the cardiometabolic risk score and triglycerides, 2) LPA was related to cardiometabolic risk score and diastolic blood pressure, and 3) MVPA was related to high-density lipoprotein cholesterol and triglycerides.  Reallocation time from SB to LPA during the non-working time on workdays was significantly associated with favorable cardiometabolic risk score changes. |
| Kurosawa (2020)^ | To examine daily patterns of SB measured objectively and sociodemographic associations in Japanese workers | N=405 | Occupation: Occupation not specified (Day-time workers)  Setting: Two cities (Koto ward, and Matsuyama) in Japan  Age: M= 51.5 [SD 7] years  -range: 40-64 years  Sex: 176 Male, 229 Female | A cross-sectional study | Device: Active style Pro HJA-350IT  Wear time: 7 consecutive days during waking hours except for water-based activities or contact sports.  Valid days: at least 4 valid days (with at least one non-workday) with 10 hours of wear; also needed a minimum of 25% valid hours of wearing time in each of the three time periods of at least three workdays and one non-workday.  Epochs: not specified  Algorithm:  - Cut points by MET: SB ≤1.5; LPA 1.5 to <3.0; and MVPA ≥3.0  -Nonwear time: at least 60 consecutive minutes of no activity with allowance for 2 min (0.9 or less METs). | Employment status, workstyle (i.e., desk-based, non desk-based) | - | - | Both workdays and non-workdays  SB was the lowest in the morning and the highest in the evening at both workdays and non-workdays.  Sociodemographic correlates differed depending on time/day:  1) Workdays, higher SB: in men, singles, higher education, those with desk workstyles, living in urban areas  -Higher SB in morning: being single, living in an urban area  -Higher SB in afternoon: higher education, those with full-time jobs, those living in the urban area  2) Non-workdays, higher SB: in men, single, those who consumed alcohol less frequently  -Higher SB in morning: single, desk workstyles, lower alcohol consumption  -Higher SB in evening: age ≥50, singles  Interaction effects with daily patterns were significant with education, workstyle, and alcohol consumption (e.g., increase in SB from afternoon to evening greater in those with lower education and non-desk-based workstyles) |
| Loprinzi (2015) | To improve understanding of the effect of shift work on PA and SB | N=1536  [By shift]  Daytime (n=1162), evening (n=68), night (n=50), rotating (n=101), and  another (n= 155) | Occupation: Occupation not specified  Setting: United States  Age: included adults ≥20 years  -Daytime shift M= 41.8 years, evening shift M= 38.8 years, Night shift M= 38.3 years, rotating shift M= 38.3 years, and another shift M= 42.6 years  Sex:  -daytime shift:  52.1% Male/ 47.2% Female  -evening shift: 56.4% Male/ 43.6% Female  -sight shift: 59.4% Male/ 40.6% Female  -rotating shift: 56.3% Male/ 43.7% Female  -another shift: 63.6% Male/ 36.4% Female | A cross-sectional study, nationally representative | Device: a wrist-worn ActiGraph AM-7164  Wear time: 7 days while awake (and removed for water activities)  Valid days: at least 4 days with 10 hours  Epochs: a 60-sec epochs  Algorithm:  - CPM: SB 0-99; LPA 100-2019; and  MVPA ≥2020  Non-wear time: period of a minimum of 60 min. of zero counts with allowance of 1-2 min of counts between 0-100 | Assessment of shift work schedule,  occupational parameters (e.g., hours worked per week) | -  · By shift:  1) daytime shift:  M= 482.2 [SD 4.0]  2) evening shift:  M= 448.2 [SD 17.2]  3) night shift:  M= 486.3 [SD 13.8]  4) rotating shift:  M= 439.9 [SD 10.3]  5) another shift:  M= 473.6. [SD 11.2] | -  · By shift:  1) daytime shift:  -LPA: M=362.4 [SD 4.7],  -MVPA: M= 28.5 [SD 0.7]  2) evening shift:  -LPA: M= 367.6 [SD 15.8]  -MVPA: M= 25.3 [SD 3.5]  3) night shift:  -LPA: M= 375.2 [SD 15.5]  -MVPA: M= 22.3 [SD 4.4]  4) rotating shift:  -LPA: M= 399.8 [SD 12.7] -MVPA: M= 29.7 [SD 1.6]  5) Another shift:  LPA: M= 376.6 [SD 11.5]  2) MVPA: M= 29.7 [SD 3.1] | Those working evening and night shifts engaged in less sustained MVPA bouts than day-time workers (p<.001), but no significant differences in MVPA.  Those working rotating shifts engaged in more LPA and less SB than day-time workers. |
| Ma (2021)^ | To identify the effects of substituting the amount of time in SB with increased PA on the body mass fat and work efficiency | N=224  55 office workers, and 169 sales and services | Occupation: Occupation not specified (full time workers from a large company)  Setting: the northeastern region of Japan  Age: M= 44.3 [SD 9.9] years  -range: 30-59 years  Sex: 180 Male/ 44 Female | A cross-sectional study | Device: a waist-worn Active Style Pro HJA-750C  Wear time: 10 working days during awake (except for bathing/swimming)  Valid days: ≤600 minutes for 4 days  Epochs: a 10-sec epochs  Algorithm:  - Cut points by MET: SB ≤1.5; LPA 1.5 to <3.0; moderate PA 3.0 to <6.0; and vigorous PA ≥ 6.0  -Non-wear time: values of 0 for 20 minutes or longer | Work efficiency (using the modified version of the World Health Organization Health and Work Performance Questionnaire Short Form) | - | - | Body fat mass was correlated with each PA: it decreased after substituting behaviors for 30 min per day from SB to vigorous PA, from LPA to vigorous PA, and from moderate PA to vigorous PA.  Greater duration in LPA had more work efficiency.  Higher work efficiency score when 30 min of SB substituted for LPA.  Highest work efficiency when vigorous PA substituted for LPA.  When 30 min of LPA substituted for VPA, work efficiency was low. |
| Neil-Sztramko (2016) | To compare PA, SB, physical fitness, and body composition in shift workers | N=3,513  Shift workers (n= 452), and  day workers (n= 3,061) | Occupation: Occupation not specified  Setting: Canada  Age:  -shift workers M=36.7 years  -day workers M= 41.7 years  Sex: 53.3% Male/ 46.7% Female  -shift workers: 53.9% Male/ 46.1% Female  -day workers: 53.2% Male/ 46.8% Female | A cross-sectional study, nationally representative | Device: a waist-worn, Actical  Wear time: 7 days during waking hours  Valid days: at least 4 valid days with ≥ 10 hours  Epochs: a 60 sec-epochs  Algorithm:  - Cut points by MET: SB< 1.0; and MVPA >3.0 METs in bouts of 10 minutes or more  -Non-wear time: at least 60 consecutive minutes of zero counts, with an allowance for one or two minutes of counts between 0 and 100 cpm | Physical fitness (the modified Canadian Aerobic Fitness Test --oxygen, and isometric handgrip strength – muscle strength), and work characteristics | M= 575.7 [CI 568.5- 582.9]  · By shift:  1) shift workers: M= 568.8 [CI 553.7- 583.9]  2) day workers: M= 576.7 [CI 569.7- 583.6] | MVPA: M=10.2 [CI 8.7- 11.8]  · By shift:  1) shift workers: MVPA M= 10.1 [CI 6.2- 13.9]  2) day workers: MVPA M= 10.2 [CI 8.7- 11.8] | SB in shift workers have less SB than day workers.  Unadjusted mean daily MVPA bouts and SB similar between shift workers and day workers.  Shift workers had lower predicted oxygen consumption than day workers in weighted analysis.  No significant differences between the occupational groups on anthropometric variables with weighted analyses. |
| Phaswana (2023) | To estimate SB and PA and to evaluate associations among SB, PA, and cardiometabolic risk factors. | N= 122 | Occupation: Office workers  Setting: University of the Wiwatersrand, and at a credit bureau company in South Africa.  Age: M= 40.2 [SD 9.3] years  Sex: 39 Male/ 83 Female | A cross-sectional study | Device: a wrist worn, AX3 monitor  Wear time: a minimum of 14 h a day for 7 consecutive days, except for water-activities.  Valid days: At least 4 days and ≥16h of wear time  Epochs: not specified  Algorithm:  - CPM: not specified  - Non-wear time: not specified | Self reported SB question | M= 180 [CI 133.0-223.3] | LPA: M=116.7 [CI 92.8-140.2]  MVPA:  M= 47.1 [CI 24.2-83.6] | Workers spent more time in LPA than MVPA.  Systolic and diastolic blood pressure were inversely associated with LPA.  No association between SB and cardiometabolic risk factors. |
| Reed (2017) | To evaluate the influence of the workplace on PA and cardiometabolic health of nurses | N= 410 | Occupation: Nurses  Setting: 14 hospitals in the Champlain local health integration network of Ontario, Canada  Age: M= 42.9 [SD 11.9] years  Sex: 23 Male/ 387 Female | A cross-sectional study, multi-center | Device: a right hip worn, ActiGraph GTX3  Wear time: 9 days during waking hours  Valid days: ≥10h of wear time, a minimum of 4 valid days  Epochs: a-15sec epochs  Algorithm:  - CPM: SB ≤150; LPA 150-2690; moderate PA 2691-6166; and vigorous PA ≥6167  - Non-wear time: at least 60 min of consecutive zeros for counts, with an allowance of up to 2 min of counts between 0-150. | International Physical Activity Questionnaire, and Perceived Workplace  Environment scale | M= 445 [SD 116] | 1) LPA:  M= 408 [SD 79]  2) moderate PA:  M= 38 [SD 18]  3) vigorous PA:  M=3  [SD 5]  4) MVPA: M=41.1 [SD 20.4] | Total SB, light-, moderate-, and vigorous-PA were calculated by shift lengths, working area, types of shifts, work status, and hospital location (Table 2 in Reed et al.’s (2018) study: page. 56).  77% of participants did not meet a physical activity guideline (150min of MVPA per week in 10-min bouts).  MVPA in bouts ≥10min was not associated with perceived workplace environment.  Nurses working 8-h shifts, fixed shifts and in urban hospitals reported better perceived workplace environment (p< .05).  Significant differences in SB and LPA were observed depending on specific workload and type of work (p<.001)  Nurses working 8-h shifts had less LPA, and greater SB, moderate PA, and MVPA than 12-h shifts (p<.05).  Nurses in administration and research positions accumulated greater SB, and less LPA than other areas except for outpatient unit (p< .05)  Fixed shifts had less LPA, and greater SB, and moderate-PA than rotating shifts (p<.05) |
| Rykov  (2020) | To examine the associations between a range of activity metrics and major modifiable biomarkers of cardiometabolic disease in a working-age population | N= 83 | Occupation:  Grouped occupations (Workers at transportation industry, a cooling plant, and a university)  Setting: 4 organizations (2 transportation companies, 1 cooling plant, and 1 university) in Singapore  Age: M= 44.3 [SD 11.9] years  -range: 22-65 years  Sex: 64 Male/ 19 Female | A cross-sectional study, data obtained from the workplace  cohort study | Device: a wrist won, Fitbit Charge 2  Wear time: consecutive 21 days except for taking a shower or charging device  Valid days: 14days with ≥18 hours (maximum of 6 missing hours per day)  Epochs: appeared to be a 60 sec epochs [addressed “in minute-by-minute intervals”]  Algorithm:  - Cut points by MET: SB ≤1.5; LPA 1.5 to <3.0; moderate PA 3.0 to ≤6.0; and vigorous PA > 6.0    - Non-wear time: not specified |  | M= 787.9 [SD 99.3] | 1) LPA:  M= 1202.9 [SD 60.2]  2) moderate PA:  M= 205.4 [SD 55.1] 3) vigorous PA:  M= 31.6 [SD 16.1] 4) MVPA M= 237.1 [SD 60.2] | Blood biomarkers and body composition biomarkers of cardiometabolic disease were associated with step-based or energy expenditure-based activity metrics measured by Fitbit: 1) participants having more steps had less triglycerides and higher high density lipoprotein cholesterol, 2) participants having more SB had a greater body mass index, and 3) participants having more vigorous PA had greater body mass index and waist circumference. |

*Note.* ANCOVA= Analyses of covariance; CI= confidential interval; CPM= cut points in counts per minute; LPA= light physical activity; M= mean; MET= metabolic equivalent; MVPA= moderate to vigorous physical activity; PA= physical activity; RCT= randomized controlled trial; SB= sedentary behavior; SD= standard deviation; ^ indicates studies reported SB and PA by specific timeline (see Supplementary Table 2); ^#^ indicates the subjective measures for SB and PA and measures related to organizational factors; ^&^ indicates showing only baseline data set in the table and having values of differences at 2-,6-,10-, and 13-months in each study.

**Table 2.**

*SB and PA by specific timeframe*   *(n=8)*

| **Reference** | **Weekdays/ working days** | | | | **Weekdays/ working days** | | **Weekends/ non-working days** | | **Definition of the time/ Notes** |
| --- | --- | --- | --- | --- | --- | --- | --- | --- | --- |
|  | **At work/ working hours** | | **At leisure/ non-working hours** | |  |  |  |  |  |
|  | **SB (min./day)**  **M [SD or 95%CI]** | **PA (min./day)**  **M [SD]** | **SB (min./day)**  **M [SD or 95%CI]** | **PA (min./day)**  **M [SD]** | **SB (min./day)**  **M [SD or 95%CI]** | **PA (min./day)**  **M [SD]** | **SB (min./day)**  **M [SD or 95%CI]** | **PA (min./day)**  **M [SD]** |  |
| Aittasalo (2017) | Baseline:  M= 298.5  [SD 81.3]  Follow up:  M= 271.3  [SD 79.2] | [Total]  Baseline:  M= 110.7  [SD 74.4]  Follow up:  M= 115.5  [SD 73.6]  [LPA]  Baseline:  M= 89.7 [SD 62.2]  Follow up:  M= 92.3 [SD 59.3]  [MVPA]  Baseline:  M= 21.0 [SD 17.8]  Follow up:  M= 23.2 [SD 20.9] | Baseline:  M= 246.9  [SD 75.7]  Follow up:  M= 255.9  [SD 74.9] | [Total]  Baseline:  M= 126.6  [SD 41.7]  Follow up:  M= 120.5  [SD 36.4]  [LPA]  Baseline:  M= 97.0  [SD 32.8]  Follow up:  M= 92.0  [SD 29.4]  [MVPA]  Baseline:  M= 29.6  [SD 20.2]  Follow up:  M= 28.5  [SD 18.3] | - | - | - | - | Working time was a classified as regular day shift, shift work without nightshifts, and other. |
| Akksilp (2023) | [Intervention]  Baseline:  M= 271  [SD 50.0]  Follow up:  M= 273  [SD 52.4]  [Control]  Baseline:  M= 275  [SD 45.2]  Follow up:  M= 276  [SD 50.4] | [Intervention]  1)LPA  Baseline:  M= 195  [SD 50.6]  Follow up:  M= 189  [SD 49.7]  2)MVPA:  M= 10.9  [SD 8.47]  Follow up:  M= 14.3  [SD 14.1]  [Control]  1)LPA:  Baseline:  M= 191  [SD 46]  Follow up:  M= 186  [SD 50.7]  2)MVPA:  M= 10.6  [SD 8.16]  Follow up:  M= 10.9  [SD 9.01] | - | - | - | - | - | - | Working hours was obtained from participants’ daily log. |
| Bergman (2018)^†^ | [Intervention]  1) sitting Baseline:  M= 275  [CI 249-301]  At 13months:  M= 271  [CI 228-314]  [Control]  1) sitting Baseline:  M= 250  [CI 225-275]  At 13months:  M= 285  [CI 244-327] | [Intervention]  1) walking  Baseline:  M= 52 [CI 47-60]  At 13months:  M= 69 [CI 56-81]  [Control]  1) walking  Baseline:  M= 50 [CI 43-56]  At 13months:  M= 48 [CI 35-59] | - | - | [Intervention]  1) sitting baseline:  M= 577  [CI 545-610]  At 13months:  M= 555  [CI 499-611]  [Control]  1) sitting baseline:  M= 540  [CI 508-572]  At 13months:  M= 565  [CI 510-619] | [Intervention]  1) LPA  Baseline:  M= 340  [CI 319-361]  At 13months:  M= 334  [CI 302-365]  2) MVPA  Baseline:  M= 59 [CI 52-66]  At 13months:  M= 46 [CI 34-57]  [Control]  1) LPA  Baseline:  M= 345  [CI 324-365]  At 13months:  M= 331  [CI 299-361]  2) MVPA  Baseline:  M= 50 [CI 43-57]  At 13months:  M= 44 [CI 32-55] | [Intervention]  1) sitting Baseline:  M= 561  [CI 523-598]  At 13months:  M= 528  [CI 454-601]  [Control]  1) sitting Baseline:  M= 541  [CI 504-578]  At 13months:  M= 507  [CI 435-579] | [Intervention]  1) LPA  Baseline:  M= 352  [CI 329-375]  At 13months:  M= 333  [CI 293-373]  2) MVPA  Baseline:  M= 68 [CI 60-76]  At 13months:  M= 51 [CI 36-67]  [Control]  1) LPA  Baseline:  M= 360  [CI 337-383]  At 13months:  M= 377  [CI 337.1-417]  2) MVPA  Baseline:  M= 62 [CI 54-70]  At 13months:  M= 52 [CI 36-67] | Authors calculated for the total time awake on weekdays and weekends, and for work time and non-work time on weekdays. |
| Fujii (2023) | - | - | - | - | Baseline:  M= 612.5 [SD 126.9]  Follow up:  M= 613.6 [SD 128.5] | 1)LPA:  Baseline:  M= 270.5 [SD 92.3]  Follow up:  M= 259.0 [SD 91.4]  2) MVPA  Baseline:  M= 68.0 [SD 25.3]  Follow up:  M= 64.1 [SD 24.4] | Baseline:  M= 539.0 [SD 150.5]  Follow up:  M= 544.9 [SD 161.3] | 1)LPA:  Baseline:  M= 276.3 [SD 94.2]  Follow up:  M= 267.8 [SD 96.8]  2) MVPA  Baseline:  M= 58.3 [SD 37.3]  Follow up:  M= 55.5 [SD 36.5] |  |
| Keown (2018)^†^ | [ActiGraph]  M= 402 [SD 72]  [ActivPAL]  Sitting/lying:  M= 384 [SD 78] | [ActiGraph]  1) LPA:  M= 84 [SD 42]  2) MVPA: M= 21 [SD 6.3]  [ActivPAL]  1) Standing:  M= 84 [SD 48]  2) Stepping: M= 42 [SD 18] | [ActiGraph]  M= 264 [SD 78]  [ActivPAL]  Sitting/lying:  M= 240 [SD 84] | [ActiGraph]  1) LPA:  M= 110.4  [SD 41.4]  2) MVPA:  M= 23.9  [SD 25.1]  [ActivPAL]  1) Standing:  M= 108 [SD 54]  2) Stepping:  M= 54 [SD 30] | [ActiGraph]  M= 666 [SD 78]  [ActivPAL]  Sitting/lying:  M= 624 [SD 96] | [ActiGraph]  1) LPA:  M= 192 [SD 54]  2) MVPA: M= 45 [SD 30.7]  [ActivPAL]  1) Standing:  M= 186 [SD 72]  2) Stepping:  M= 96 [SD 36] | [ActiGraph]  M= 546 [SD 108]  [ActivPAL]  Sitting/lying:  M= 492 [SD 132] | [ActiGraph]  1) LPA:  M= 252 [SD 72]  2) MVPA: M= 34 [SD 29.5]  [ActivPAL]  1) Standing:  M= 234 [SD 96]  2) Stepping:  M= 102 [SD 48] | During a total 7-day period, for workdays, non-workdays, work hours on a workday, and non-workhours (i.e., leisure) on a workday were calculated. |
| Kitano (2022) | M= 346.4  [SD 72.2] | 1) LPA:  M= 112.0  [SD 23.3]  2) MVPA:  M= 21.5 [SD 4.5] | M= 389.1  [SD 40.5] | 1) LPA:  M= 173.5  [SD 18.1]  2) MVPA:  M= 59.7 [SD 6.2] | - | - | M= 632.8  [SD 43.9] | 1) LPA:  M= 321.9  [SD 22.4]  2) MVPA:  M= 59.3 [SD 4.1] | Working time is from 9am to 5pm. |
| Kurosawa (2020) | - | - | - | - | M= 507.3  [SD 140.2] | 1) LPA:  M= 352.4  [SD 126.7]  2) MVPA:  M= 77.4  [SD 47.7] | M= 510.7  [SD 127.5] | 1) LPA:  M= 313.2  [SD 109.5]  2) MVPA:  M= 56.7  [SD 36.0] |  |
| Ma (2021) | - | - | - | - | M= 408.2  [SD 78.3] | 1) L PA:  M= 22.6  [SD 63.9]  2) moderate PA:  M= 82.4  [SD 21.2]  3) vigorous PA:  M= 4.7 [SD 8.5] |  |  |  |

*Note.* CI= confidential interval; LPA= light physical activity; M= mean; MVPA= moderate to vigorous physical activity; SB= sedentary behavior; SD= standard deviation; PA= physical activity; and ^†^ indicates that SB was measured by activPAL, and PA levels were measured by ActiGraph.
